# Multidisciplinarity is vital to pandemic management and preparedness

**DOI:** 10.1186/s12982-026-02267-5

**Published:** 2026-06-18

**Authors:** Martyn Pickersgill

**Affiliations:** School of Population Health Sciences, University of Edinburgh, Edinburgh, UK

**Keywords:** Pandemic preparedness, COVID-19, Multidisciplinarity, Humanities and social sciences, Science advice, Health policy

## Abstract

Governments around the world sought to address the Covid-19 pandemic through a range of engagements with various kinds of experts. At the same time, some disciplinary perspectives were privileged over others. For instance, while calls were made during the pandemic for more social science expertise on advisory panels, formal mechanisms for integrating insights from these disciplines were often more limited than would have been ideal. Some governments are now undertaking inquiries to assess national responses to Covid-19, including in relation to how knowledge and evidence was leveraged during the pandemic. In the UK, the official Covid-19 inquiry is ongoing, yet reports for the first two ‘Modules’ have been published and bear scrutiny for relevant insights. Module 2 remarks on the importance of different perspectives in tackling the SARS-CoV-2 virus and the wider effects of infection on society. Moving forward, to prepare for future pandemics full engagement across a wider range of disciplines – including across the humanities and social sciences – is essential.

## Introduction

During and after the Covid-19 pandemic, many questions were posed to national governments and the institutions of global health. What role should non-pharmaceutical interventions play in the management of infectious disease? Why do some forms of governance generate varying kinds of public contestation across different contexts? Whose authority and expertise are relevant to public health decision-making? In relation to the latter, for instance, calls were made to enhance engagement with the humanities and social sciences. A range of countries have set up inquiries and similar initiatives to consider pandemic responses, addressing to different degrees some of the key questions that emerged over 2020-22 (although the extent to which such deliberations will ultimately improve accountability and policy learning remain unclear) [1].

Within the UK, the Covid-19 Inquiry commenced in 2022 and is ongoing. Undertaken as a series of ‘Modules’, November 2025 saw the publication of Module 2: a two-volume report on ‘Core decision-making and political governance’ [2, 3]. Perhaps unsurprisingly, these volumes make sobering reading. They detail a range of issues regarding the management of the pandemic, including in relation to scientific advice mechanisms. The report also includes several instructive observations and recommendations; for example, clarifying structures of decision-making as well as public communications in the case of future emergencies.

## The importance of multidisciplinarity

One matter that risks evading sufficient attention is the Inquiry’s emphasis on the significance of multidisciplinary expertise within advisory bodies. Module 2 Volume II, for instance, notes that it is:

important that experts are drawn from a diverse range of disciplines, backgrounds and experience. The incorporation of multiple voices, including those with dissenting views, helps to build sufficient challenge into the advisory process and to guard against ‘groupthink’ (p23) [3].

However, we know that around the world such inputs were not always apparent during the Covid-19 pandemic. As noted above, despite appeals at the time to integrate more humanities and social scientific contributions into advisory mechanisms, engagement with such expertise was more limited than it should have been [4, 5]. The incorporation of additional experts from disciplines like science and technology studies, the medical humanities, and linguistics into national and global advisory boards and planning initiatives could have enhanced pandemic responses. This could have been achieved through, for instance, translation of a greater variety of evidence, data, and understandings into policy.

In particular, a wider range of experts could have helped to refocus attention on how individuals and populations respond to and negotiate pandemic law, policy, and guidance beyond specific behaviours; i.e., through social practices. By this term, sociologists and other social scientists mean the routinised and emergent forms of action constituted by societal traditions, institutions, and diverse forms of knowledge. Attention to how social practices are enacted and become reworked through societal perturbations (including law and regulation, alongside Covid-19 per se) would have generated a far richer picture of social life during the pandemic. Doing so could in turn have helped to frame policy decisions, interventional roll-out, and generated more granular and multi-dimensional evaluations of how different interventions operated across social settings.

As the UK Covid-19 Inquiry notes, the “inclusion of experts from a more diverse range of backgrounds [on advisory groups] might have helped to challenge orthodox scientific thinking” (pp24-25) [3]. Humanities and social science scholars would have provided great benefit in this respect. Sociologists, for instance, have considerable expertise with regards to how social practices and dynamics shape how interventions instantiate and are (not) engaged with in societies. They are also highly skilled in parsing the negative ramifications from ostensibly laudable interventions, and the sometimes erroneous assumptions undergirding these. Such insights include unveiling the specificities of the accumulated distrust that communities might have in relation to healthcare based on prior harms [6]. This knowledge matters if public health institutions are to not only seek to enhance trust, but vitally to help demonstrate their trustworthiness (such as through acknowledging instances where healthcare has previously harmed particular communities, and developing contemporary interventions with such history as essential context).

Asking awkward questions, and presenting unexpected or unwanted findings, does not always make academic experts (such as those in the humanities and social sciences) popular in some advisory contexts. This is particularly when their expertise sits outside the usual boundaries of the disciplines involved in advisory mechanisms addressing a domain like infectious disease. It does, though, make them essential as a source of constructive and supportive challenge; for instance, to public health practitioners and healthcare policymakers with regards to the management of epidemics and pandemics.

## Conclusion

As the UK Covid-19 inquiry implies, data-driven and conceptually rich inputs from across disciplines are needed to enhance health policy and care. This includes through enabling better understandings of the complex social worlds within which public health strategies are actioned and interventions implemented. Integrating insights from multiple forms of expertise can be difficult. However, frameworks to support multidisciplinary policy decision-making are being innovated, and their implementation could provide benefit to advisory bodies aiming to engage a broader range of specialisms [7, 8].

Currently, a number of pandemic preparedness initiatives are being rolled-out internationally. Going forward, it is essential that these fully embrace the perspectives and expertise of a wider set of disciplines than have traditionally been deployed or emphasised to address public health emergencies [8, 9]. The humanities and social sciences must be key among these.

## Data Availability

No datasets were generated or analysed during the current study.
